# Steroid-Refractory Warm Autoimmune Haemolytic Anaemia Complicating Ulcerative Colitis in Pregnancy: A Case Report and Management Considerations

**DOI:** 10.7759/cureus.104937

**Published:** 2026-03-09

**Authors:** Jimmy William, Conor Lahiff, Maria Couto

**Affiliations:** 1 Haematology, Mater Misericordiae University Hospital, Dublin, IRL; 2 Gastroenterology, Mater Misericordiae University Hospital, Dublin, IRL

**Keywords:** autoimmune-hemolytic anemia, immunosuppression, pregnancy, rituximab, ulcerative colitis (uc)

## Abstract

Autoimmune haemolytic anaemia (AIHA) arising in pregnancy is an exceptionally rare condition, and its concurrence with active ulcerative colitis (UC) presents considerable diagnostic and therapeutic complexity. We describe a 30-year-old primigravida who presented at 12 weeks' gestation with severe symptomatic anaemia (haemoglobin 4.4 g/dL), serologically confirmed warm AIHA (strongly positive direct antiglobulin test, IgG-mediated), and concurrent gastrointestinal haemorrhage attributable to an acute flare of UC. High-dose corticosteroid therapy failed to achieve a durable response, and the patient developed steroid-refractory disease characterised by recurrent haemolytic episodes necessitating repeated red cell transfusion. Infliximab was initiated for refractory colitis but was rendered ineffective by the development of anti-drug antibodies. Subsequent therapeutic escalation comprised intravenous immunoglobulin, rituximab, azathioprine, and darbepoetin alfa; vedolizumab was later introduced to achieve intestinal remission. Following rituximab administration, haemoglobin stabilised progressively, transfusion dependence resolved, and haemolysis indices normalised, notwithstanding persistent direct antiglobulin test positivity. By 32 weeks' gestation, haemoglobin had risen to above 10 g/dL with complete clinical remission of haemolysis. Delivery was expedited at 35 weeks' gestation by caesarean section owing to superimposed pre-eclampsia; no haemolytic relapse was observed in the peripartum period. The neonate was admitted to the neonatal intensive care unit for three weeks due to respiratory distress syndrome but demonstrated no evidence of haemolysis or neonatal alloimmune or autoimmune complications. This case illustrates that UC-associated, steroid-refractory AIHA in pregnancy, whilst rare, is amenable to successful management through timely escalation to biologic and immunosuppressive therapy, judicious use of erythropoiesis-stimulating agents to minimise transfusion burden, and close multidisciplinary collaboration between haematology, gastroenterology, and maternal-foetal medicine, with favourable maternal and neonatal outcomes achievable.

## Introduction

Autoimmune haemolytic anaemia (AIHA) is a rare condition that, whilst generally manageable, becomes potentially life-threatening when it occurs in pregnancy, where autoantibody-mediated red cell destruction may also affect the foetus. Management is particularly complex given the competing priorities of maternal and foetal safety, especially during the teratogenically vulnerable first trimester. Whilst most cases are idiopathic, secondary AIHA may arise in the context of autoimmune diseases-including inflammatory bowel disease (IBD)-infections, or lymphoproliferative disorders [1]. Ulcerative colitis (UC)-associated AIHA is uncommon but can present with significant severity during disease flares [2].

The overlap between immune activation, pregnancy physiology, and treatment toxicity presents unique therapeutic dilemmas. Ideally, patients with complex autoimmune conditions such as UC should undergo periconceptional counselling to optimise disease control, stratify risk, and select pregnancy-compatible therapies before conception [3]; in this case, however, failure to adequately evaluate and follow up on the underlying intestinal inflammation prior to pregnancy meant that disease activity was unrecognised and uncontrolled at the time of presentation.

We report a case of steroid-refractory warm AIHA in a pregnant patient with a severe UC flare requiring combination immunosuppressive and biologic therapy. We discuss the relative contributions of haemolysis and haemorrhage to the clinical picture, the risk-benefit considerations for each therapeutic intervention, and the multidisciplinary approach required in such cases.

## Case presentation

A 30-year-old primigravida presented to the Emergency Department at nine weeks' gestation with symptomatic anaemia and eight episodes of rectal bleeding daily. She had a known history of UC, previously treated with corticosteroids, azathioprine, and infliximab; however, infliximab had been discontinued several months prior to presentation following the development of mild haemolysis, after which a watch-and-wait approach was adopted, leaving her without active disease-modifying therapy at the time of presentation. Physical examination was unremarkable except for pallor.

Laboratory investigations revealed profound haemolytic anaemia with a haemoglobin of 4.4 g/dL, markedly elevated lactate dehydrogenase (LDH), reticulocytosis, indirect hyperbilirubinaemia, undetectable haptoglobin, and a strongly positive (4+) direct antiglobulin test (DAT) reactive for anti-IgG only (warm thermal amplitude), with negative anti-C3d, anti-C3c, and anti-IgM (Table 1).

**Table 1:** Laboratory investigations on first presentation. NRBC: nucleated red blood cell; INR: international normalised ratio; eGFR: estimated glomerular filtration rate; ALT: alanine transaminase; gamma-GT: gamma-glutamyl transferase

Test	Value	Reference range	Units
Full blood count			
Haemoglobin (Hb)	4.4	12.0-16.0 (F)	g/dL
Haematocrit (HCT)	0.129	0.36-0.46 (F)	L/L
White blood cells (WBC)	9.85	4.0-11.0	×10⁹/L
Lymphocytes (LYMP)	1.12	1.0-4.0	×10⁹/L
Platelets (PLT)	359	150-400	×10⁹/L
NRBC	0.1	0.0	×10⁹/L
Coagulation			
INR	1.15	0.9-1.2	-
Prothrombin time (PT)	12.8	9.0-13.0	secs
Electrolytes & renal			
Sodium	137	133-146	mmol/L
Potassium	3.3	3.5-5.3	mmol/L
Chloride	108	98-107	mmol/
Urea	2.5	2.5-7.8	mmol/L
Creatinine	42	45-84 (F)	µmol/L
eGFR	>60	>60	mL/min/1.73 m²
Liver function			
Bilirubin	41	3-21	µmol/L
Alkaline phosphatase	69	30-130	IU/L
ALT	6	7-56	IU/L
Gamma-GT	12	6-42 (F)	IU/L
Albumin	36	35-50	g/L
Bone profile			
Calcium	2.05	2.20-2.60	mmol/L
Corrected calcium	2.14	2.20-2.60	mmol/L
Phosphate	1.01	0.8-1.5	mmol/L
Magnesium	0.76	0.7-1.0	mmol/L
Haematinics & inflammation			
Vitamin B12	368	200-900	ng/L
Serum folate	15.1	4.6-18.7	µg/L
CRP	70	<5	mg/L
Ferritin	1,008	13-150 (F)	µg/L
Transferrin saturation	18.1	20-50	%
Iron	9.0	11-29 (F)	µmol/L
Transferrin	1.90	2.0-3.6	g/L

Antibody studies demonstrated no erythrocyte antigen specificity, reacting with all panel cells including Rh-null cells, and no alloantibodies were identified. The patient's extended red cell phenotype was O RhD+ C+ E− c+ e+ Cw− M+ N+ S+ s+ K+ k+ Fy(a−) Fy(b+) Jk(a+) Jk(b−) Do(a+) Do(b+) Vel+. Peripheral blood film demonstrated polychromasia and spherocytosis with fewer than 5% schistocytes. Urinalysis was unremarkable. A comprehensive infectious and virological screen, including cytomegalovirus (CMV), Epstein-Barr virus (EBV), parvovirus B19, COVID-19, and tuberculosis, was negative for active infection. A general autoimmunity screen revealed a positive connective tissue disease (CTD) screen (ratio 1.60; reference range 0.03-0.70), which incorporates antigens including U1RNP, SS-A/Ro, SS-B/La, Scl-70, Jo-1, and Sm; however, reflex testing for anti-dsDNA and ENA-specific antibodies was negative. HIV, hepatitis B, and hepatitis C serologies were negative. Haemoglobin high-performance liquid chromatography (HPLC) and serum protein electrophoresis were normal. Flow cytometry for paroxysmal nocturnal haemoglobinuria was negative. Abdominal ultrasound was unremarkable.

A multidisciplinary team (MDT) comprising maternal medicine, gastroenterology, and haematology reached consensus to initiate high-dose oral prednisolone at 1 mg/kg/day (65 mg) alongside folic acid supplementation. Transfusional support was provided targeting a haemoglobin of ≥8 g/dL using extended phenotype-matched and CMV-negative red cell units. Aspirin and prophylactic low-molecular-weight heparin (LMWH) were commenced. Following two weeks of inpatient treatment, the patient demonstrated partial haematological recovery and initial clinical improvement and was discharged on prednisolone 50 mg/day with a planned slow taper of 10 mg every two weeks.

**Figure 1:**
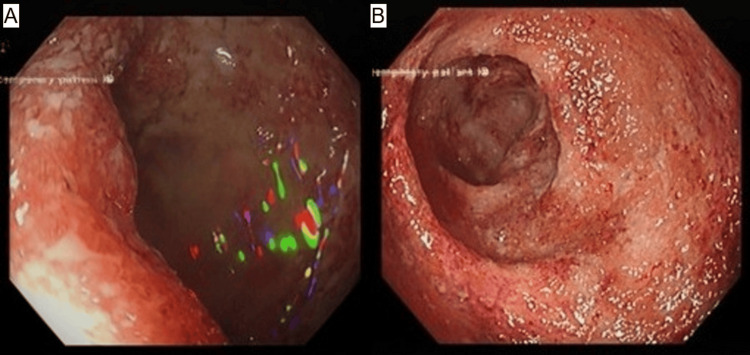
(A, B) Flexible sigmoidoscopy demonstrating marked erythema, mucosal oedema, ulceration, and friability with complete loss of normal haustral folds. The endoscopic appearance was consistent with severe proctitis (Mayo endoscopic subscore 3), in keeping with an acute ulcerative colitis flare.

However, approximately one week after discharge, whilst on prednisolone 50 mg/day, the patient relapsed with worsening fatigue, nausea, and bloody diarrhoea (eight episodes per day). The decline in haemoglobin at this stage was likely multifactorial, reflecting both ongoing autoimmune haemolysis and significant gastrointestinal blood loss from the UC flare. Flexible sigmoidoscopy demonstrated severe proctitis (Figure 1). Infliximab was added to corticosteroid therapy, and darbepoetin alfa (30 mcg weekly) and valaciclovir prophylaxis were initiated. Although this led to transient clinical stabilisation and discharge, she was readmitted shortly thereafter with a third episode of acute decompensation characterised by a precipitous fall in haemoglobin and brisk extravascular haemolysis in the context of ongoing, though reduced, bloody diarrhoea, without any identifiable infectious trigger. Of note, the patient had experienced a mild, self-limiting haemolytic episode during a prior course of infliximab two years before conception, which had not required medical intervention.

Given the severity and persistence of haemolysis, UC-related immune dysregulation was considered the most plausible driver, and therapy was escalated across both specialties. Prednisolone was dose-adjusted to 1 mg/kg based on current weight, intravenous immunoglobulin (IVIg, 1 g/kg/day) was administered over two days, and azathioprine was introduced at 1 mg/kg/day. The patient concurrently developed gestational diabetes mellitus, requiring subcutaneous insulin. Due to ongoing haemoglobin instability, azathioprine was up-titrated to 2.5 mg/kg/day and darbepoetin alfa increased to 50 mcg weekly. In view of refractory haemolysis, rituximab was commenced at 375 mg/m² weekly for four doses, completing treatment at 19 weeks' gestation. On the gastroenterological side, anti-infliximab antibodies were detected at a titre of >10 ng/mL with a subtherapeutic infliximab trough level of <3 µg/mL, confirming immunogenic loss of response; this prompted a switch to vedolizumab for ongoing UC management. Rituximab was initiated one week after the final infliximab infusion and one week prior to vedolizumab, at a point when serum infliximab levels remained detectable. Co-trimoxazole prophylaxis was added in view of the degree of cumulative immunosuppression. Additional supportive measures included folic acid, intramuscular vitamin B12, and intravenous iron.

**Figure 2:**
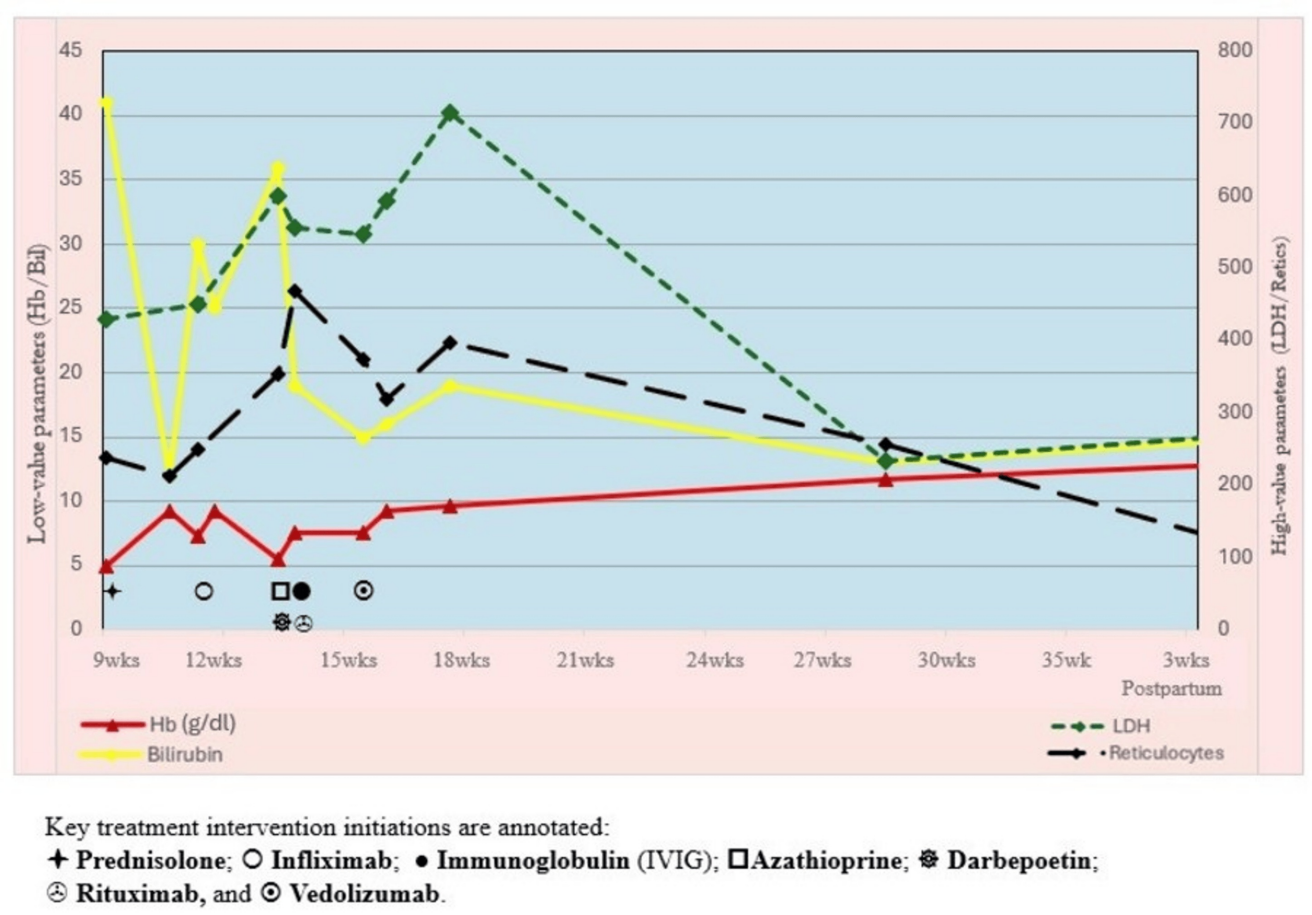
Dual Y-axis trends in haematologic parameters during and after pregnancy under immunosuppressive therapy. Haemoglobin (Hb), bilirubin, lactate dehydrogenase (LDH), and reticulocyte counts are shown over time.

Following the introduction of rituximab and vedolizumab, haemoglobin and indirect bilirubin began to stabilise (Figure 2). Although haemolysis markers remained abnormal-with persistently elevated LDH, unquantifiable haptoglobin, and a DAT that remained positive at a reduced titre (anti-IgG 2+)-gastrointestinal bleeding resolved completely following initiation of vedolizumab, administered as per standard induction protocol via intravenous infusion at week 0, week 2, and week 6, followed by maintenance subcutaneous injections every two weeks thereafter. A total of 13 red cell transfusions were administered, all prior to the initiation of rituximab and resolution of gastrointestinal haemorrhage. There was no clinical or biochemical evidence of intravascular haemolysis at any point during the admission.

By 32 weeks' gestation, the patient was clinically stable with improved functional capacity and no further gastrointestinal bleeding. Haemoglobin exceeded 10 g/dL on a sustained basis, permitting discontinuation of darbepoetin alfa, whilst azathioprine was maintained at 2.5 mg/kg/day. Serum bilirubin normalised, though haptoglobin remained unquantifiable and the DAT persistently positive. In the event of relapse, therapeutic plasma exchange had been identified as a contingency intervention; splenectomy and colectomy had also been discussed but were deemed inadvisable given the gestational stage and clinical risk.

Foetal surveillance was consistently reassuring, with no evidence of intrauterine growth restriction and normal amniocentesis findings. Pre-eclampsia was identified at 34 weeks, and the patient underwent a planned caesarean section at 35 weeks' gestation. No peripartum haemolytic relapse occurred, and no clinically significant haemoglobin decline was observed. Aspirin and prophylactic LMWH were continued until six weeks postpartum. The patient remained free of infection throughout pregnancy. The neonate was admitted to the neonatal intensive care unit (NICU) for three weeks requiring supplemental oxygen for pneumonia and respiratory distress syndrome but demonstrated no serological or clinical evidence of haemolysis.

## Discussion

AIHA complicating pregnancy is rare, with an estimated incidence of fewer than one per 100,000 pregnancies [1-11]. This case is notable not only for the severity of haemolysis and its association with UC but also for its presentation in the first trimester-a period in which therapeutic decisions carry particular consequences given the vulnerability of concurrent organogenesis.

Periconceptional planning is strongly recommended for patients with complex autoimmune conditions such as UC [3]. Establishing disease remission on pregnancy-compatible medications prior to conception reduces the risk of flares during organogenesis and enables timely multidisciplinary engagement. In this case, the patient had not been receiving maintenance UC therapy for several months before conception, and AIHA was identified late and in a severely decompensated state. This highlights the real-world consequences of missed periconceptional counselling and the clinical trajectory that may follow.

A critical and recurrent issue throughout this case was the dual contribution of haemolysis and gastrointestinal haemorrhage to the patient's anaemia. At initial presentation and during subsequent relapses, haemoglobin decline reflected both ongoing red cell destruction-evidenced by elevated LDH, reticulocytosis, indirect hyperbilirubinaemia, and undetectable haptoglobin-and active gastrointestinal blood loss from UC. This distinction carries direct clinical implications: high-dose corticosteroids, aspirin, and LMWH-all prescribed in this patient-have the potential to exacerbate mucosal bleeding. Transfusion thresholds and requirements must therefore be interpreted within this dual context, and haemoglobin recovery attributed to the combined effect of haemolysis suppression and haemostasis. Notably, the eventual cessation of transfusion requirements coincided with both the introduction of rituximab and the resolution of rectal bleeding following vedolizumab, rendering it impossible to attribute haemoglobin recovery to either intervention alone.

Regarding the immunological characteristics of the warm autoantibody, the DAT was strongly positive for IgG (4+) with warm thermal amplitude, and negative for IgM and complement components (anti-C3d and anti-C3c). IgG autoantibodies in warm AIHA are predominantly of the IgG1 and IgG3 subclasses, which bind Fc receptors on macrophages most efficiently and carry the highest haemolytic potential [1,12]. IgG1 and IgG3 also cross the placenta most efficiently via FcRn-mediated transport, a mechanism that becomes particularly active beyond 20 weeks' gestation. Conversely, IgG2 and IgG4 subclasses exhibit lower haemolytic potential and cross the placenta less efficiently. IgG subclass determination was not performed in this case and is acknowledged as a limitation; such information would have informed assessment of foetal haemolytic risk and the relative urgency of rituximab administration before the period of maximal transplacental IgG transfer. The negative neonatal DAT and absence of neonatal haemolysis observed may reflect placental exclusion of the specific subclass present, neutralisation through rituximab-mediated B-cell depletion prior to 20 weeks, or intrinsic neonatal compensatory erythropoietic capacity-distinctions that cannot be confirmed without subclass data.

The indications and risk-benefit profiles of each therapeutic intervention warrant individual consideration, summarised here alongside their pregnancy safety classifications. Prednisolone (FDA Category C) was initiated as first-line therapy for warm AIHA per international guidelines [11]. First-trimester exposure carries a small risk of orofacial clefting, with earlier studies reporting an odds ratio of approximately 1.7 for cleft lip with or without palate, though larger, more recent population-based studies have not confirmed this association, suggesting the absolute risk-if present-is extremely small (<0.2%) [13]. Additional recognised risks include gestational diabetes, which occurred in this patient, hypertension, preterm birth, and low birth weight, particularly with prolonged use [14]. These risks were deemed acceptable given life-threatening haemolysis. Aspirin (FDA Category C/D near term) and LMWH (FDA Category B) were commenced for pre-eclampsia prophylaxis and thromboprophylaxis, respectively. LMWH does not cross the placenta and carries no established foetal risk. Both agents carry a theoretical risk of exacerbating gastrointestinal bleeding, a tradeoff explicitly considered by the MDT. Infliximab (FDA Category B) was introduced to target UC-driven immune activation perpetuating haemolysis. PIANO registry data confirm no significant increase in congenital malformations, spontaneous abortion, preterm birth, or low birth weight with anti-TNF exposure in IBD pregnancies, though neonatal drug levels may persist up to six months, warranting avoidance of live vaccines in the neonate during this period [15]. Its failure due to immunogenic loss of response prompted a switch to vedolizumab. Vedolizumab (FDA Category B) is a gut-selective anti-α4β7 integrin monoclonal antibody that restricts lymphocyte trafficking to the intestinal mucosa, resulting in minimal systemic immunosuppression-a particularly favourable property in pregnancy. In this case, it addressed intestinal inflammation as a key driver of ongoing haemolysis whilst limiting broader immune perturbation. The European CONCEIVE study demonstrated no significant increase in miscarriage risk or congenital anomalies compared to anti-TNF-exposed and biologic-unexposed IBD controls [16], and PIANO registry data similarly show no increase in adverse maternal or foetal outcomes with vedolizumab exposure [15]. It is endorsed by the European Crohn’s and Colitis Organisation (ECCO) guidelines as a preferred biologic in pregnant patients with IBD. Data specific to its use in concurrent AIHA in pregnancy are currently unavailable, and this case contributes to the limited literature in this area. IVIg (FDA Category C) provided rapid transient immunomodulation as a bridge to rituximab, with no established teratogenic risk in published obstetric data. Azathioprine (FDA Category D), despite its classification, is broadly accepted in pregnancy where the benefit outweighs the risk, with PIANO data showing no significant increase in congenital anomalies [15]. Rituximab (FDA Category C) was used off-label for refractory AIHA; when administered before 20 weeks, as in this case, foetal risk is minimised due to limited transplacental IgG transfer before this gestational window. The principal concern is transient neonatal B-cell lymphopenia, reported in approximately 12% of neonates in the global rituximab safety database, with cytopenias typically resolving within six months [17]. Darbepoetin alfa (FDA Category C) was introduced to stimulate erythropoiesis and reduce transfusion-related alloimmunisation risk [4].

Published reports of AIHA complicating pregnancy describe complete haematological responses in approximately 65% of cases, with maternal complication rates of around 15%-including pre-eclampsia and preterm delivery-and adverse foetal or neonatal events in approximately 22% of cases, including respiratory distress, preterm birth, and neonatal AIHA [2,5-10]. This case encountered several of these outcomes: pre-eclampsia identified at 34 weeks, caesarean delivery at 34 + 6 weeks, and neonatal respiratory distress requiring NICU admission, reflecting the substantial morbidity associated with this condition even when haematological control is ultimately achieved.

This case also illustrates the diagnostic challenge of distinguishing UC-associated AIHA from drug-induced immune haemolytic anaemia secondary to infliximab. Infliximab can rarely precipitate haemolysis through IgG- or IgM-mediated destruction of drug-coated red cells [18-20]. The history of mild, self-limiting haemolysis during prior infliximab exposure two years before conception raises this possibility; however, the haemolysis encountered in the current admission was considerably more severe, correlated temporally with UC flare activity rather than with drug administration, and ultimately responded to UC-directed therapy. The concurrent development of anti-infliximab antibodies further complicates attribution but also appropriately redirected the therapeutic strategy towards vedolizumab.

## Conclusions

This case demonstrates that UC-associated, steroid-refractory warm AIHA in pregnancy, though exceptionally rare, is amenable to successful management when escalation to combination immunosuppressive and biologic therapy is pursued early and decisively. The therapeutic turning point was the combination of rituximab-administered prior to 20 weeks' gestation to minimise transplacental IgG transfer-and vedolizumab. In this infliximab-refractory case, vedolizumab proved to be the pivotal intervention: its gut-selective mechanism directly targeted the intestinal immunological trigger perpetuating haemolysis, whilst its favourable pregnancy safety profile made it uniquely suitable in this clinical context. This underscores that in AIHA driven by IBD, effective control of the intestinal trigger is as integral to haematological recovery as direct immunosuppression of the haemolytic process itself.

Key lessons include the critical importance of periconceptional counselling in complex autoimmune conditions, the value of early therapeutic escalation when corticosteroid-refractory disease is established, the role of erythropoiesis-stimulating agents in minimising transfusion-related alloimmunisation risk, and the importance of IgG subclass determination to inform foetal risk assessment. Clinicians managing similar cases should consider vedolizumab early in the treatment algorithm when anti-TNF therapy fails or is contraindicated, particularly in pregnancy, where gut-selective immunosuppression offers a compelling risk-benefit advantage. Favourable maternal and neonatal outcomes are achievable but depend on sustained multidisciplinary collaboration and meticulous reassessment of the therapeutic risk-benefit balance as pregnancy advances.
